# Survival outcomes of double- and triple-sequential targeted therapy in patients with metastatic renal cell carcinoma: a retrospective comparison

**DOI:** 10.18632/oncotarget.21926

**Published:** 2017-10-19

**Authors:** Sung Han Kim, Yoon Seok Suh, Jung Kwon Kim, Jae Young Joung, Ho Kyung Seo, Kang Hyun Lee, Jinsoo Chung

**Affiliations:** ^1^ Center for Prostate Cancer, Research Institute and Hospital of National Cancer Center, Goyang, Korea

**Keywords:** renal cell carcinoma, metastasis, sequential, targeted therapy, survival

## Abstract

**Objective:**

To evaluate the progression-free survival (PFS) and overall survival (OS) in patients with metastatic renal cell carcinoma (mRCC) treated with double- and triple-sequence targeted therapy (TT) using tyrosine kinase inhibitors (TKIs) and mammalian target of rapamycin inhibitors (mTORi).

**Materials and Methods:**

Records of 292 patients with mRCC, treated with TT between January 2005 and July 2015, were analyzed retrospectively. Kaplan-Meier and log-rank analyses were used to calculate and compare the total PFS (tPFS) and OS when patients underwent double- or triple-TT using TKIs or mTORi.

**Results:**

Eighty-one (27.7%) patients who underwent second-line TT were enrolled; 30 (10.3%) of whom underwent third-line TT. The tPFS and OS of double-TT using TKI-mTORi (5.4 and 30 months, respectively) were significantly better compared with TKI-TKI (0.3 and 2 months) or mTORi-TKI (2 and 6 months) (p <0.001). For triple-TT, the tPFS and OS of TKI-mTORi-TKI (22.8 and 25 months, respectively) were significantly superior compared with those for TKI-TKI-mTORi (4 and 9 months) (p <0.05).

For patients with intermediate-risk according to the Heng or Memorial Sloan-Kettering Cancer Center risk models, TKI-mTORi was associated with a significantly longer tPFS and OS compared with TKI-TKI [expect for OS in the Heng group (p = 0.086)]. For the triple TT group, TKI-mTORi-TKI resulted in improved tPFS and OS compared with TKI-TKI-TKI or TKI-TKI-mTORi (p <0.05).

**Conclusion:**

In patients with mRCC, sequential administration of TKI-mTORi led to a significantly superior tPFS compared with any other TT sequence. By contrast, OS did not differ significantly according to TT sequence.

## INTRODUCTION

The advent of multiple targeted therapies (TT) for the treatment of metastatic renal cell carcinoma (mRCC) has renewed hope for increasing the therapeutic response rate, slowing disease progression and improving survival outcomes. Complete responses to treatment are rare, and patients eventually progress, requiring subsequent lines of therapy for disease control [1-3]. Following first-line tumor progression, individualized sequential therapy has become the standard treatment [4-6]. As the number of TTs used for second-, third-, and fourth-line therapies increase, so too do the potential sequential combinations in which they can be administered. For patients with mRCC, the optimal sequence to obtain maximum clinical benefit and improve progression-free survival (PFS) and overall survival (OS) is unknown.

Tyrosine kinase inhibitors (TKI) of the vascular epithelial growth factor (VEGF)-receptor and mammalian target of rapamycin inhibitors (mTORi) are the major drug classes used for mRCC treatment. For their anticancer activity, these classes utilize distinct pathways with minimal cross-resistance. Therefore, alternating TT sequentially can improve therapeutic efficacy. The most commonly employed TT sequences are TKI-TKI-mTORi and TKI-mTORi-TKI [2, 5-9], but there is limited evidence for the optimal sequential TT use for mRCC, especially in Asian patients [5, 6, 9]. This study aimed to compare the survival outcomes of patients who underwent sequential treatment using double- or triple-TT, with or without immunotherapy (ITx). Outcomes were reported as total PFS (tPFS) and OS, according to drug treatment sequence and risk, as classified using the initial prognostic criteria of the Memorial Sloan-Kettering Cancer Center (MSKCC) [10] and the International Metastatic Renal Cell Carcinoma Database Consortium (Heng criteria) risk models [11].

## RESULTS

### Baseline patient characteristics

Between 2005 and 2015, the records for 292 patients with mRCC were included. Eighty-one patients were included in the final analysis. Baseline patient characteristics are shown in Table 1. The median age was 55 years, and patients were predominantly male. Second-, third-, and fourth-line TT were administered to 81 (27.7%), 30 (10.3%), and 9 (3.1%) patients, respectively. Nephrectomy and metastasectomy rates were 28.4% and 34.6%, respectively. The overall median treatment durations for double- and triple-TT were 30.2 (5.3–66.7) months and 37.8 (8.0–83.8) months, respectively.

**Table 1:** Baseline patient characteristics (N=81)

	N(%) or Median (min-max)
Age (years)	55 (30-76)
gender (Male/ Female)	64/ 17 (79/ 21)
Nephrectomy/metastasectomy	22/ 28 (28.4/ 34.6)
Heng favorable risk	15 (18.5)
Intermediate risk	60 (74.1)
Poor risk	6 (7.4)
MSKCC favorable risk	18 (22.2)
Intermediate risk	56 (69.1)
Poor risk	7 (8.6)
Pathologic/clinical T, T2, T3, T4	23/12/35/11 (13.5/28.4/14.8/43.2)
N0, N, Nx	20/19/42 (24.7/23.5/51.8)
M	54 (66.7)
Fuhrman nuclear grade 1/2/3/4/unknown	8/23/24/3/23 (9.9/28.4/29.6/3.7/28.4)
Histology Clear cell/ Non-clear cell/unknown	68/7/6 (84/8.6/7.4)
Second line ITx/TKI/mTORi (N=81)	10/35/36 (12.3/43.2/44.5)
Third line ITx/TKI/mTORi (N=30)	5/16/9 (16.7/53.3/30)
Forth line TT	9 (11.1)
Double Sequential TT	
TKI-mTORi	39 (48.1)
TKI-TKI	30 (37.1)
TKI-ITx	10 (12.3)
mTORi-TKI	2 (2.5)
Double sequence response-RECIST	
CR/PR/SD/PD	0/6/48/27 (0/7.4/59.3/33.3)
Triple sequential TT	
TKI-TKI-TKI	1 (3.3)
TT-TT-ITx	9 (30.0)
ITx-TT-TT	8 (26.7)
TKI-mTORi-TKI	6 (20.0)
TKI-TKI-mTORi	6 (20.0)
Triple sequence response-RECIST	
CR/PR/SD/PD	0/5/17/8 (0/16.6/56.7/26.7)
Treatment duration of Second/Third-line TT(Month)	3.1 (1-66.7)/5.0 (1-47.1)
Overall median duration of double/triple sequential TT (months)	30.2 (5.3-66.7)/37.8 (8-83.8)
Total PFS of Double/Triple sequential TT (Month)	10.2 (1-74.4)/17.8 (3.5-83.8)
OS of Double/Triple sequential TT (Months)	30.0 (21.1-38.8)/40.0 (18.4-61.6)
Survival/death	14/ 67 (17.3/82.7)

TT, targeted therapy; ITx, immunotherapy; TKI, tyrosine kinase inhibitor; mTORi, mammalian target of rapamycin inhibitor; CR, complete remission; PR, partial response; SD, stable disease; PD, progressive disease; PFS, progression-free survival; OS, overall survival

### Survival durations according to double- or triple-TT

The tPFS durations were 10.2 and 17.8 months for patients who underwent double-TT or triple-TT, respectively. The OS durations were 30.0 and 40.0 months for those who underwent double-TT or triple-TT, respectively. Fourteen (17.3%) patients remained alive at study completion.

### Survival durations according to sequential double-TT

**Figure 1:**
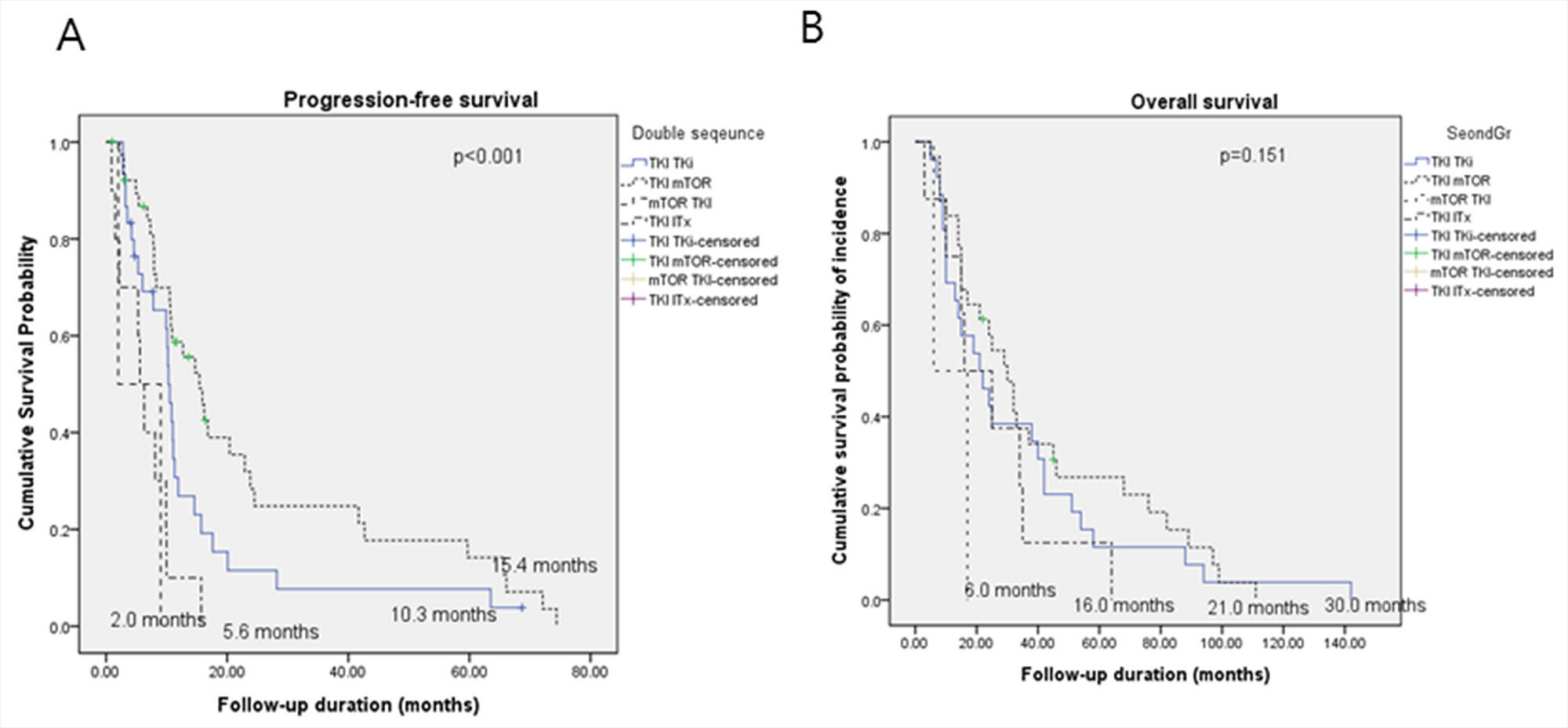
Kaplan-Meier curves of **(A)** progression-free survival and **(B)** overall survival according to double sequential targeted therapy.

TKI-mTORi use (n = 39, 48.1%; tPFS, 15.4 months; OS, 30 months) resulted in superior tPFS durations (p <0.001) compared with other double-TT regimens as follows: TKI-TKI (n = 30, 37%; tPFS, 10.3 months; OS, 21 months), mTORi-TKI (n = 2, 2.5%; tPFS, 5.6 months; OS, 16 months), and TKI-ITx (n = 10, 12.3%; tPFS, 2 months; OS, 6 months). However, the differences in OS durations were not significant (p = 0.151) (Figure 1).

### Survival durations according to sequential triple-TT

**Figure 2:**
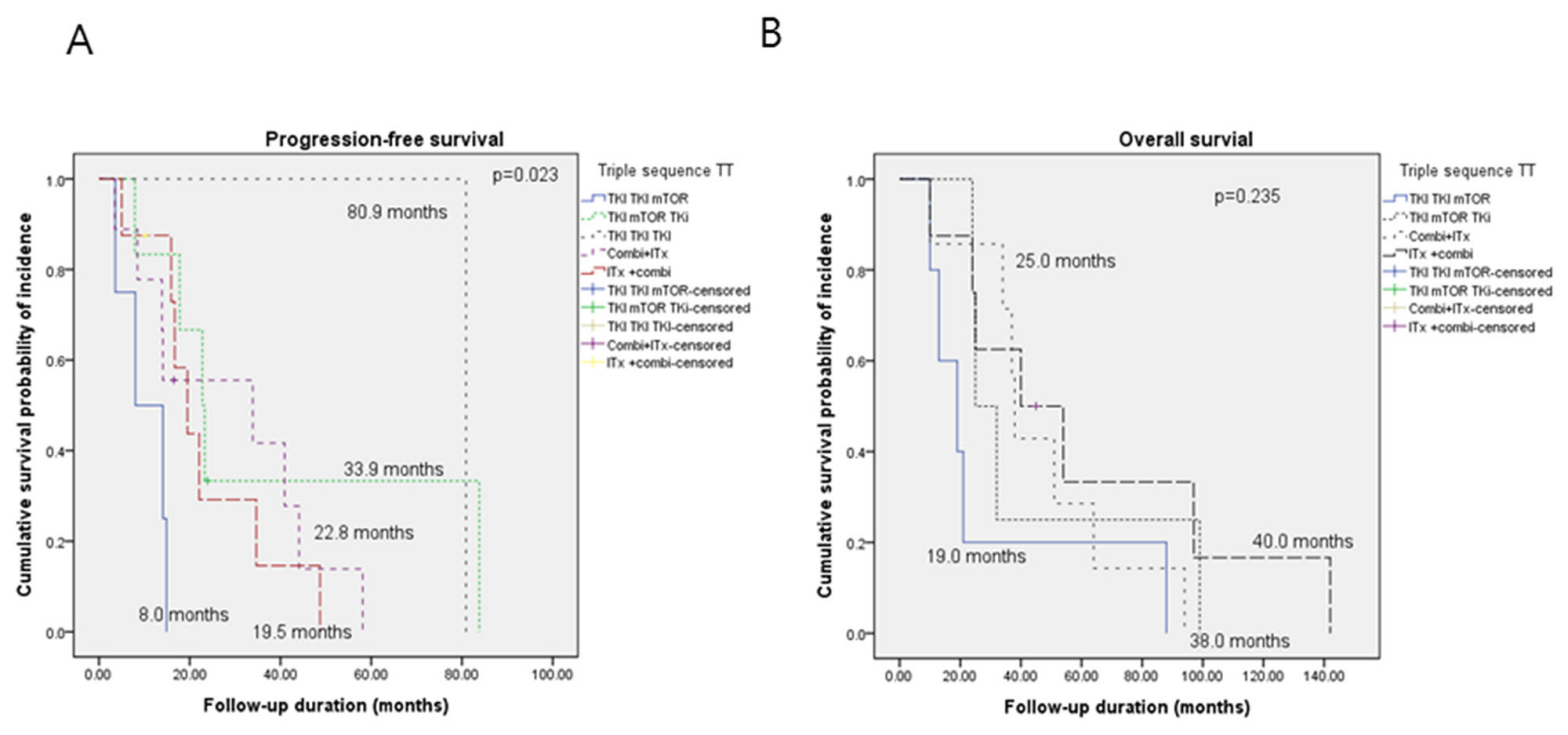
Kaplan-Meier curves of **(A)** progression-free survival and **(B)** overall survival according to triple sequential targeted therapy.

tPFS durations (p = 0.023), but not OS durations (p = 0.235), were significantly different according to the triple-TT regimens administered, as follows: TT-TT-ITx (n = 9, 29%; tPFS, 33.9 months; OS, 38.0 months), TKI-TKI-TKI (n = 1, 3.2%; tPFS, 80.9 months; OS, not available), TKI-mTORi-TKI (n = 6, 19.4%; tPFS, 22.8 months; OS, 25 months), TKI-TKI-mTORi (n = 7, 22.6%; tPFS, 14.1 months; OS, 19 months), and TT-ITx-TT (n = 8, 25.8%; PFS, 8.0; OS, 19.0 months) (Figure 2).

An additional subgroup analysis of PFS for sequential triple-TT to evaluate all five potential triple-TT combinations did not show any significant differences (p = 0.083, data not shown), except for the comparison of TKI-TKI-mTORi (2.2 months) and TKI-mTORi-TKI (5.1 months; p = 0.022, Supplementary Figure 1).

### Survival in patients according to double- or triple-TT according to risk

For the double-TT groups, when stratified by risk classifications, only patients with favorable- or intermediate-risk had significantly different tPFS and OS durations (p <0.05, except for the MSKCC intermediate-risk groups whose OS durations were not significantly different [p = 0.086]) (Table 2). For the triple-TT groups, significant differences were noted for tPFS in the MSKCC and Heng intermediate risk groups (p <0.05), whereas there were significant differences for OS in the MSKCC favorable (p = 0.038) and Heng intermediate (p = 0.002) risk groups. In patients with a poor risk, similar to those in the double-TT groups, there were no significant differences in survival durations (p >0.05; Table 3).

**Table 2:** Comparison of total progression-free survival and overall survival of double sequence according to the prognostic models of the MSKCC and Heng criteria

MSKCC/Heng criteria	Favorable (mos., 95% CI)	Intermediate (mos., 95% C. I.)	Poor (mos., 95% C.I.)
PFS (p-value)	<0.001/0.002	0.009/0.003	0.768/ 0.636
TKI-mTORi	42.7 (17.8-67.6)	15.4 (8.8-22.0)/ 15.4 (6.6-24.2)	2.9 (1.7-6.6)
TKI-TKI	10.2 (1-24.0)/ 10.2 (3.5-16.9)	10.5 (9.6-11.4)/ 10.8 (9.8-11.8)	5.3 (2.4-12.3) / 4.6 (3.2-12.7)
TKI-ITx	5.6 (1-12.1)	8.1 (1-17.3)/ 8.1 (1.0-15.3)	5.3 (3.3-8.5)/ NA
mTORi -TKI	NA	2.0 (1-12.4)	NA
OS (p-value)	0.024/ 0.046	0.086/ 0.048	0.737/ 0.863
TKI- mTORi	115 (104.7-125.3)/ 125 (1-275.4)	33 (19.6-46.4)	8^*^
TKI-TKI	67 (1-159.1)/ 115 (104.7-125.3)	16 (19.6-46.4)	8 (4.1-11.9) / 9 (4.1-13.9)
TKI-ITx	14.0 (1-62.8)	18.0 (7.2-28.8)	16^*^
mTORi -TKI	NA	5.0 (1-21.3)	NA

^*^. only one patient’s clinical outcome

C.I, confidence interval; PFS, progression-free survival; OS, overall survival

**Table 3:** Comparison of total progression-free survival and overall survival of triple sequences according to prognostic models of the MSKCC and Heng criteria

MSKCC/ Heng criteria	Favorable (mos., 95% CI)	Intermediate (mos., 95%C.I.)	Poor (mos., 95%C.I.)
PFS (p-value)	0.183/ 0.248	0.041/ 0.012	0.317/ 0.273
TT-TT-ITx	33.9 (1-74.5) / 8.5 (1-56.5)	14.1 (1-43.1) / 33.9 (1-74.5)	NA
ITx-TT-TT	22.1 (17.9-26.3) / 22.1 (9.3-61.5)	15.9 (4.4-27.4) / 16.7 (13.2-20.2)	5^*^
TKI-mTORi-TKI	17.8^*^	23.3 (1-63.2)	7.9^*^
TKI-TKI-mTORi	NA	8.0 (1.0-15.0)	14.1^*^
OS (p-value)	0.030/0.090	0.078/ 0.006	NA/ 0.317
TT-TT-ITx	67 (35.0-99.0) / 47.0 (9.6-162.4)	40.0 (1-79.2) / 49 (19.7-63.3)	NA
ITx-TT-TT	76 (1-154.4) / 111.0 (38.6-175.4)	29 (1-71.1) / 53 (6.9-99.1)	NA/ 10^*^
TKI-mTORi-TKI	25^*^	33 (18.6-47.4)	NA
TKI-TKI-mTORi	88^*^	12.0 (7.2-16.8)	NA/ 21^*^

^*^, only one patient’s outcome

C.I, confidence interval; PFS, progression-free survival; OS, overall survival

### Survival in patients on sequential double-TT according to risk

**Figure 3:**
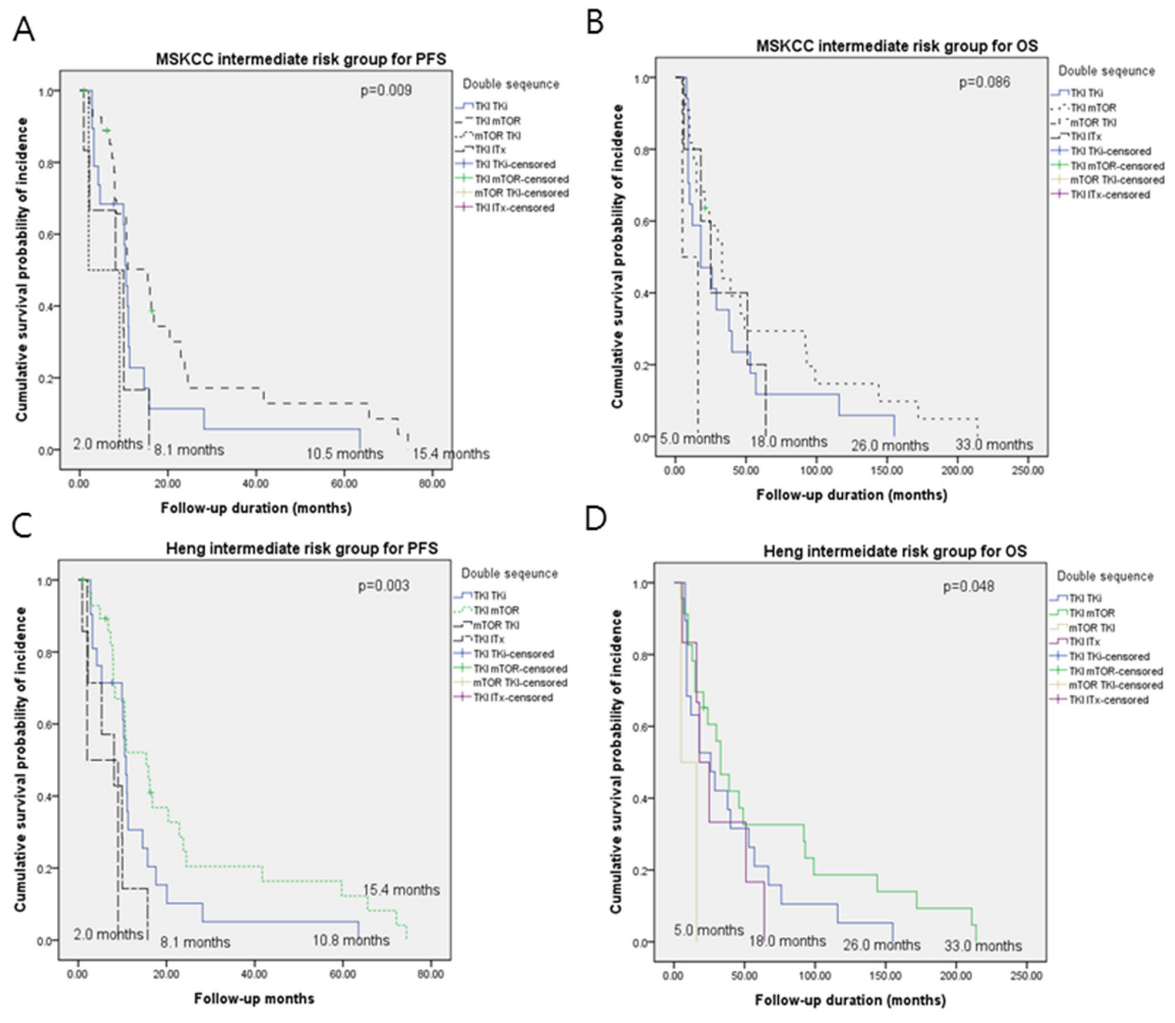
Kaplan-Meier curves of **(A, C)** progression-free survival and **(B, D)** overall survival according to triple sequential targeted therapy in mRCC patients with intermediate MSKCC and Heng risks.

For the 56 patients categorized as MSKCC intermediate-risk, tPFS durations for TKI-mTORi, TKI-TKI, TKI-ITx, and mTORi-ITx groups were 5.4, 10.5, 8.1, and 2.0 months (p = 0.009), and the OS durations were 33, 26, 18, and 5.0 months (p = 0.086), respectively. For the 50 patients categorized as Heng intermediate-risk, tPFS durations for TKI-mTORi, TKI-TKI, TKI-ITx, and mTORi-ITx were 15.4, 10.8, 8.1, and 2 months, and OS durations were 33, 26, 18, and 5 months, respectively (Figure 3).

### Survival in patients on sequential triple-TT according to risk

For MSKCC intermediate-risk patients (n = 15), the tPFS durations for TT-TT-ITx, TKI-mTORi-TKI, ITx-TT-TT, and TKI-TKI-TKI groups were 23.3, 15.9, 14.1, and 8 months, respectively. For the same patients, the OS durations for TT-TT-ITx, TKI-mTORi-TKI, ITx-TT-TT, and TKI-TKI-mTORi were 33, 29, 40, and 12 months (p >0.05), respectively.

**Figure 4:**
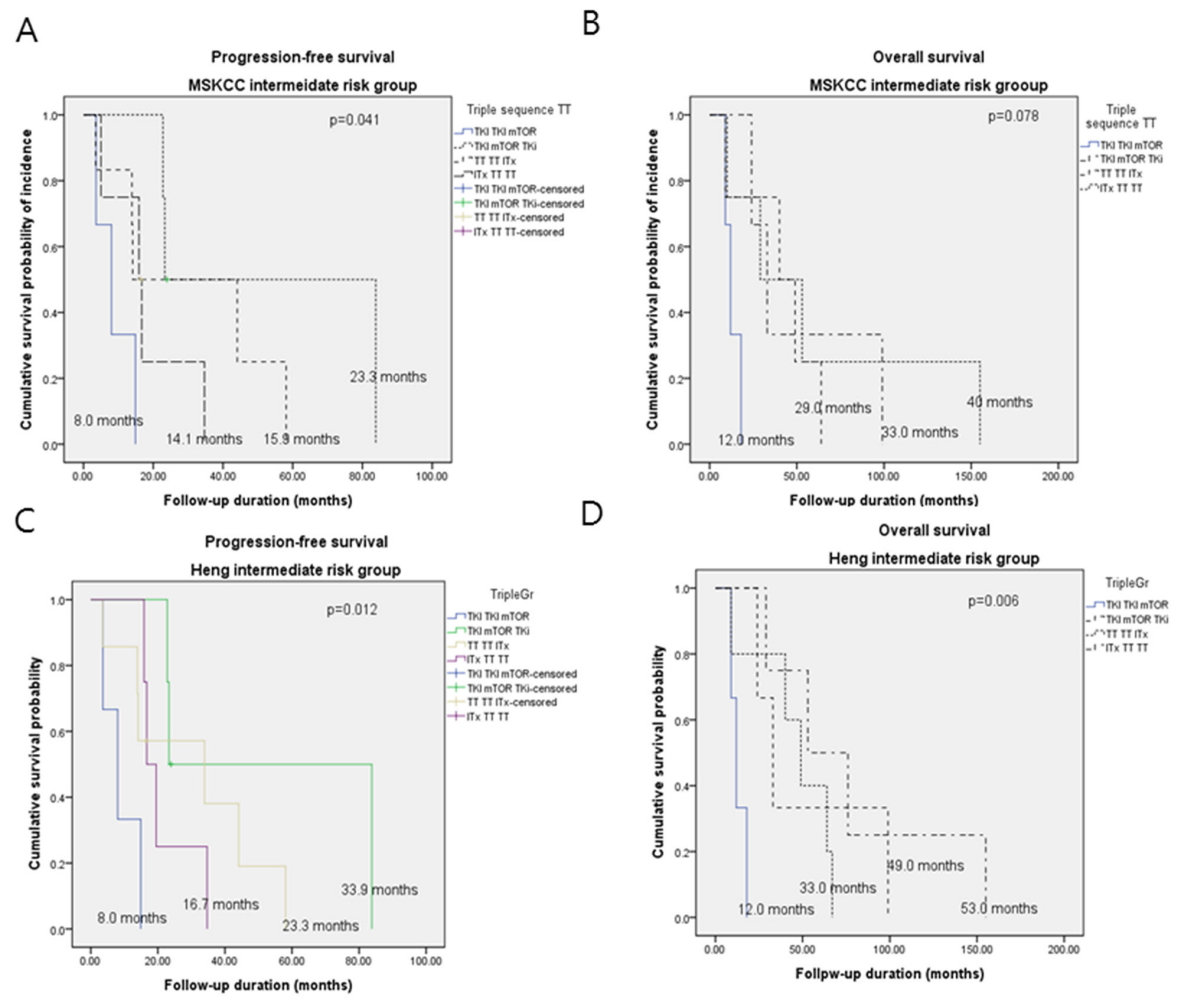
Kaplan-Meier curves of **(A, C)** progression-free survival and **(B, D)** overall survival according to triple sequential targeted therapy in mRCC patients with intermediate MSKCC and Heng risks.

For Heng intermediate-risk patients (n = 16), the tPFS durations for TKI-mTORi-TKI, TT-TT-ITx, ITx-TT-TT, and TKI-TKI-mTORi were 33.9, 23.3, 16.7, and 8 months, respectively, while the OS durations for TKI-mTORi-TK, ITx-TT-TT, TT-TT-ITx, and TKI-TKI-mTORi were 53, 49, 33, and 12 months, respectively (p <0.05) (Figure 4).

## DISCUSSION

TT has considerably improved prognosis in patients with mRCC with an approximate doubling of median OS since the immunotherapy era [12]. Multiple randomized, controlled, clinical trials of single TT have established the objective therapeutic response rate, PFS, and OS. For maximum clinical benefit, TT should be used sequentially and tailored to the patient’s needs. However, despite multiple guidelines with established positions on the sequential use of TT, the optimal combination is unclear [5, 6].

For mRCC, the underlying concepts of sequential TT are to prevent treatment resistance and augment anti-tumor activity. Tumor cells can develop treatment resistance via upregulation of pro-angiogenic signals (e.g., placental growth factor or fibroblast growth factor) or by increased expression of protective pericytes that express the platelet-derived growth factor receptor. These crucial mechanisms can be more efficiently inhibited using multi-targeted TKIs, compared with administration of everolimus or bevacizumab alone [3, 13]. Following progression during TKI administration, another concept is to switch to an mTORi, which operate via a different pharmacodynamic profile to augment antitumoral effects.

In the present study, the tPFS and OS of each drug sequence was compared, and patients who were treated with TKI-mTORi, had a significantly longer tPFS, and a numerically higher (although not statistically significant) OS compared with those who were administered TKI-TKI (Figure 1, Supplementary Figure 2). This finding is similar to that of an observational meta-analysis conducted by Heng et al., which compared the PFS and OS of second-line mRCC treatment using an mTORi versus a TKI [4, 14]. They showed that second-line use of an mTORi was associated with significantly prolonged survival, compared to the second-line use of a TKI. Based on this evidence, the optimal treatment sequence dilemma of whether to shift to an mTORi or to persevere with angiogenesis inhibition (VEGF-TKI), is one of the main unresolved investigational issues for patients with mRCC [15, 16].

Current guidelines for mRCC are of limited value in selecting TT, because of heterogeneity between patients, an almost complete absence of evidence-based data on sequential therapy, and the lack of predictive biomarkers for the TT approved for RCC [5, 6]. According to evidence-based guidelines, most patients with mRCC are initially treated with TKI, except patients with an unfavorable prognosis who might be prescribed temsirolimus [17]. The optimal treatment choice following the failure of first-line TKI remains a matter of debate since head-to-head, prospective clinical studies comparing the efficacy between a TKI and an mTORi in patients who have failed initial TKI therapy have not been conducted [17, 18]. Therefore, dissemination of data regarding practice-oriented TT use in patients with mRCC is desirable and of increasing importance.

The choice between either a TKI or an mTORi for the subsequent, second-line TT should be based on toxicity profile, efficacy, and tolerability, as well as potential cross-resistance between the first and second-line TT. The AXIS clinical trial, which compared the effectiveness of axitinib versus sorafenib in advanced RCC, did not show absolute cross-resistance between TKIs [19]; however, the sequential use of two pharmacodynamically similar TKIs could be associated with cumulative gastrointestinal side effects.

Escudier et al. [17] suggested that rapid therapy switching should be avoided for patients with slow disease progression, with a switch performed when progression occurred. Such an approach would keep other therapeutic options available for as long as possible [20]. For rapidly progressing disease, however, a rapid switch should be considered as early as possible. In the cases where tumor response is mixed, e.g. stabilization in one lesion but progression in another, and when there is evidence of new metastases, isolated progressive tumors or metastases should be treated with local surgery and radiotherapy [6]. However, in the case of relevant progression, a switch in systemic therapy is necessary. Furthermore, a switch is obligatory if there is unacceptably high toxicity, or if the conservative management of adverse events fails.

For third-line sequential TT, in the present study, TKI-mTORi-TKI doubled OS compared with TKI-TKI-mTORi. This finding is dissimilar to that of Iacoville et al. who indicated that TKI-TKI-mTORi resulted in superior outcomes compared with TKI-mTORi-TKI [8]. Such discrepancies could be due to the heterogeneity of data sources, confounding factors, selection bias due to the lack of randomization, and the small number of cases in the present study. In the triple-TT group, there were only 2 patients with favorable- and poor-risk classifications, and the remainder were classified as intermediate-risk. The proportion of poor-risk patients in Iacoville et al.’s study (6%) was similar to that in the present study (8.6%). However, the proportion of favorable-risk patients was much higher in that study (46%) compared with the present study (22.2%) suggesting that the sequential combinations including a second-line mTORi might be associated with more favorable outcomes. By contrast, and in agreement with the present study, Heng et al. found that TKI-mTORi-TKI had superior outcomes, similar to the finding that TKI-mTORi had significantly better outcomes compared to use of two successive TKIs [14].

A subanalysis comparing all combinations of third-line TTs showed that the PFS associated with triple TT was significantly lower in patients treated with TKI-TKI-mTORi compared to that in those treated TKI-mTORi-TKI. These findings suggest that third-line TT using a TKI might be better tolerated and associated with improved quality of life compared with a third-line mTORi [21]. Such superiority of TKI-mTORi-TKI compared with TKI-TKI-mTORi might be because the use of pazopanib as a third-line TKI might inhibit not only VEGF pathways but other alternative angiogenesis pathways [22, 23]. Lastly, the cross-resistance between similar TKIs and the differential modes of action might affect the superiority of TKI-mTORi-TKI [24].

In the present study, ITx was also used as a second- and third-line drug. The TT-TT-ITx and ITx-TT-TT groups had favorable treatment outcomes compared with other triple-TT groups. Recent reports have raised the possibility of a potential immunomodulatory effect of TT [25, 26], and data underlining the effect of mTORi on anticancer immunity have been presented [27]. Recent randomized clinical trials with new target agents, in the second- or third-line setting, demonstrated better efficacy than second-line everolimus for TKI-failed mRCC patients [28]. OS values after treatment with cabozantinib (21.4 months), and lenvatinib with everolimus (14.6 months) were significantly better compared with everolimus alone (18.8 and 5.5 months, respectively) [28]. An immune checkpoint inhibitor, called nivolumab has shown improvement of OS compared with everolimus in the second- and third-line setting [29]. These new agents are likely to further change the approach to sequential and combinational TT treatment in mRCC.

Subgroup analyses based on prognostic risk showed variable prognostic outcomes between the double- and triple-TT. The discordance in survival outcomes, according to the risk models, in patients with an intermediate risk, might be explained by the small numbers of patients, which could have an impact on the risk assessment variables used to determine intermediate risk between the two models [20]. The MSKCC model was developed during the immunotherapeutic era for patients undergoing ITx and has been shown to have limitations, and a weaker predictive ability for survival in patients treated using TT, compared with the Heng model.

The present study has limitations. It was a retrospective study in selected patients, who were healthy enough to be treated using triple-TT. The relatively low numbers of patients included for each TT combination can be seen as hypothesis-generating, but does not allow any definitive conclusions to be made regarding the optimal TT sequence. Other factors such as patient preference, comorbidities, tumor burden, symptoms, and different prognostic models need to be considered when making treatment decisions. Also, the treatment cost and availability, depending on the health insurance system of each country, should be considered. Future large-scale, prospective studies with a greater number of patients undergoing each treatment type are needed to gain a better insight into the optimal TT sequence in patients with mRCC.

In conclusion, the present study did show that TKI-mTORi sequential therapy resulted in significantly superior tPFS compared with other double-TT therapies, whereas OS was not dependent on the combination of sequential TT. The combination of sequential TT with ITx also showed favorable tPFS and OS.

## MATERIALS AND METHODS

### Ethics statement

This retrospective study was approved by the Institutional Review Board (IRB) of the National Cancer Center (IRB No. NCC206-0262). The need for written consent was waived. Patient data were anonymized and de-identified before analysis. Study procedures were performed in accordance with the ethics of the Declaration of Helsinki.

### Eligibility criteria

The records of patients with mRCC, treated between January 2005 and July 2015 with a TT, were collected from a prospective RCC database. Patients with non-measurable disease, or no follow-up records, were excluded. Initial risk classifications based on the MSKCC [10] and Heng risk criteria [11] were used to predict response to double- and triple-TT.

Fuhrman nuclear grade and the Tumor-Node-Metastases staging (Union for International Cancer Control, 2009) classification were used to evaluate RCC pathologically [30]. Treatment response was measured according to the Response Evaluation Criteria in Solid Tumors (RECIST, version 1.1) [31] using computed tomography or magnetic resonance imaging performed according to local procedures (between every 8-12 weeks). Disease progression was defined as a ≥20% increase in the longest diameter, as per RECIST, or the development of any new metastatic lesion.

The choice of TT sequence was decided by the treating clinician (JC), guided by the patient’s pathology and coverage by National Insurance [32]. Standard dosage reductions, in cases of toxicity, were managed by the treating physician. TT was classified into two groups, based on the mechanism of action: TKIs (axitinib, pazopanib, sorafenib, and sunitinib) or mTORis (everolimus and temsirolimus). In addition, some patients underwent interferon-alpha ITx during second- or third-line treatment. All agents were administered in accordance with manufacturers’ instructions.

### Statistical analysis

The tPFS and OS durations following double- or triple-TT were the primary outcomes. Differences and associations between the baseline characteristics were examined using the Chi-square test, Fisher’s exact test, and the Kruskal-Wallis test, as appropriate. The tPFS was defined as the sum of PFS time for each double- and third-line TT, from the date of TT initiation to the date of disease progression, from any cause. OS was defined as the period from treatment initiation to death by any cause or to the last contact with the patient. Statistical analyses of tPFS and OS for each combination of TT were evaluated according to the MSKCC and Heng risk criteria using Kaplan-Meier curves with the log-rank test. All results were considered statistically significant if the two-sided p values were < 0.05 using SAS 9.2 software (SAS Institute Inc., Cary, NC, USA).

## SUPPLEMENTARY MATERIALS FIGURES



## Abbreviations

mRCC: metastatic renal cell carcinoma
TKI: tyrosine kinase inhibitor
mTORi: mammalian target of rapamycin inhibitor
TT: targeted therapy
ITx: immunotherapy
PFS: progression-free survival
tPFS: total progression-free survival
OS: overall survival
