# Supplementary material for: Survival outcomes of double- and triple-sequential targeted therapy in patients with metastatic renal cell carcinoma: a retrospective comparison
Source: Oncotarget. 2017 Oct 19;8(59):100056–65. doi: 10.18632/oncotarget.21926 (PMC5725002; doi:10.18632/oncotarget.21926)
Supplement: Supplementary file 1 [file oncotarget-08-100056-s001.pdf]

Survival outcomes of double- and triple-sequential targeted therapy in patients with metastatic renal cell carcinoma: a retrospective comparison

SUPPLEMENTARY MATERIALS

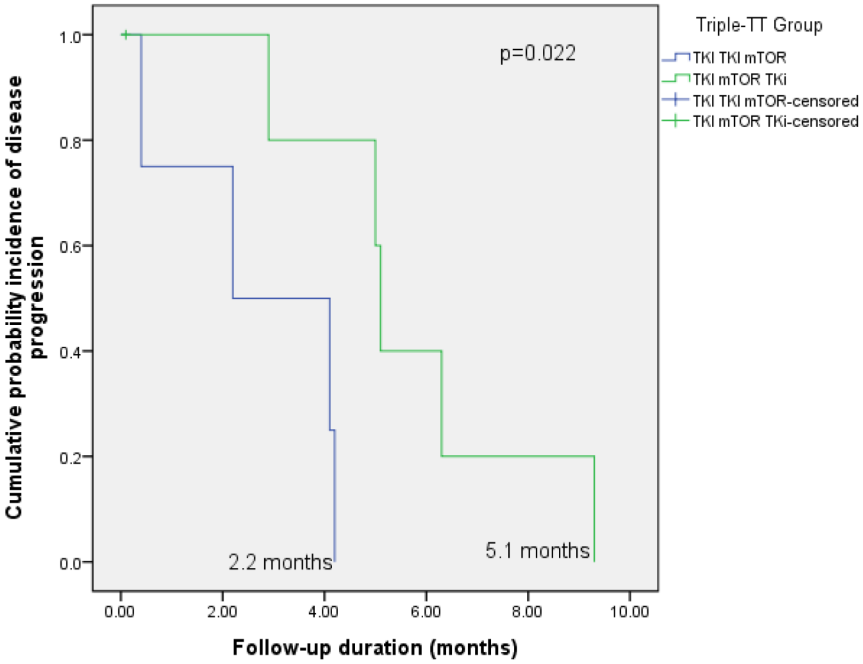

Supplementary Figure 1: Comparison of third-line progression-free survival of sequential triple-targeted therapy groups between TKI-TKI-mTORi and TKI-mTORi-TKI.

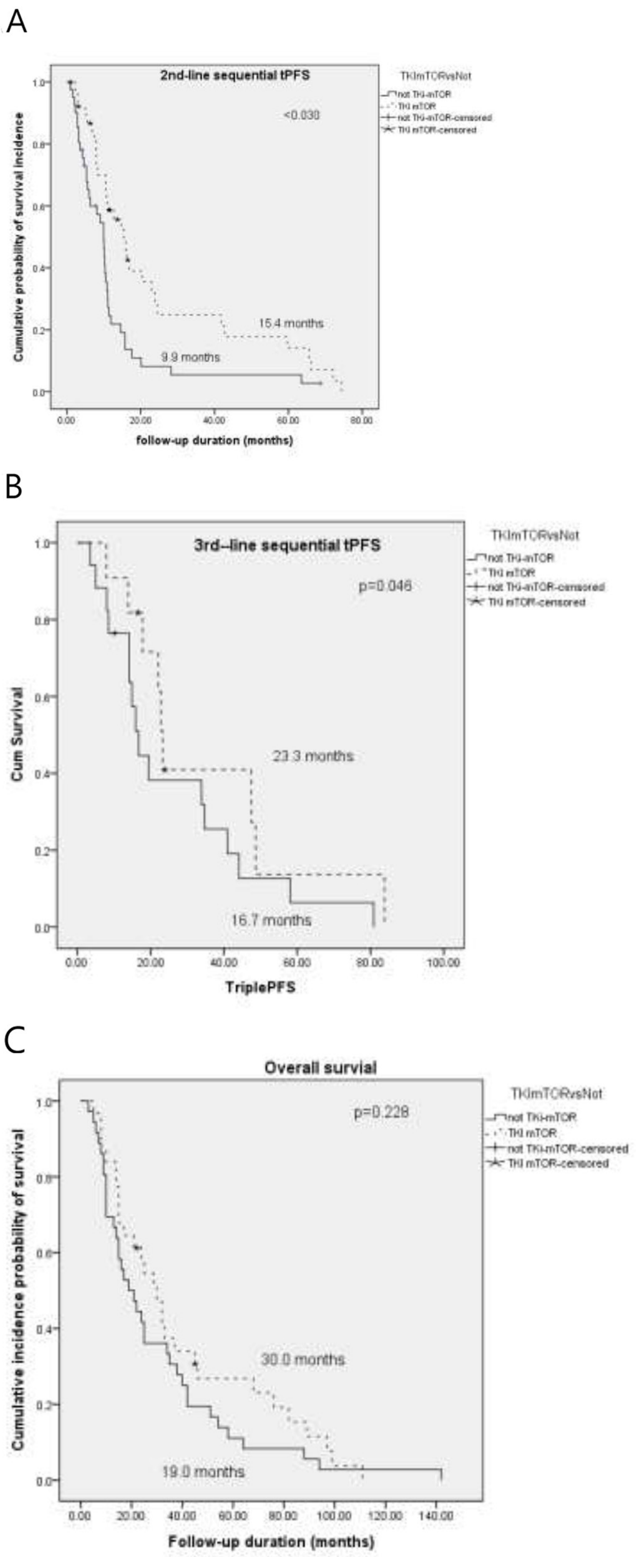

Supplementary Figure 2: Comparative survivals of total progression-free survival in **(A)** 2nd-line and **(B)** 3rd-line sequential therapy and **(C)** overall survival between TKI-mTORi and non-TKI-mTORi sequential therapy groups.
